# Chromatin Architectural Changes during Cellular Senescence and Aging

**DOI:** 10.3390/genes9040211

**Published:** 2018-04-16

**Authors:** Luyang Sun, Ruofan Yu, Weiwei Dang

**Affiliations:** Huffington Center on Aging, Department of Molecular and Human Genetics, Baylor College of Medicine, Houston, TX 77030, USA; Luyang.Sun@bcm.edu (L.S.); Ruofan.Yu@bcm.edu (R.Y.)

**Keywords:** chromatin architecture, aging, cellular senescence, Hi-C

## Abstract

Chromatin 3D structure is highly dynamic and associated with many biological processes, such as cell cycle progression, cellular differentiation, cell fate reprogramming, cancer development, cellular senescence, and aging. Recently, by using chromosome conformation capture technologies, tremendous findings have been reported about the dynamics of genome architecture, their associated proteins, and the underlying mechanisms involved in regulating chromatin spatial organization and gene expression. Cellular senescence and aging, which involve multiple cellular and molecular functional declines, also undergo significant chromatin structural changes, including alternations of heterochromatin and disruption of higher-order chromatin structure. In this review, we summarize recent findings related to genome architecture, factors regulating chromatin spatial organization, and how they change during cellular senescence and aging.

## 1. Introduction

Aging is a complex biological process that involves multiple cellular and molecular functional declines, including cellular senescence, genomic instability, and epigenetic alterations [1]. Numerous studies have illustrated that the eukaryotic 3D genome is highly dynamic. Significant chromatin structural changes occur during physiological aging and senescence, from alterations in the nuclear envelope and the structure of chromosome territories within the nucleus to changes in nucleosome positioning and histone modifications [2,3,4]. These chromatin changes include global histone loss, the alternation of epigenetic landscapes, chromatin spatial interaction changes, loss of heterochromatic regions, and large-scale chromatin rearrangements. Different models and cell types have been used to study the changes associated with aging and senescence. Some of the aging regulatory mechanisms are conserved among eukaryotic species, such as the target of rapamycin (TOR) signaling-related nutrient-sensing pathway [5]. However, it is interesting to note that different models may have specific chromatin changes. For example, although global loss of heterochromatic regions and reduction of repressive histone modification markers occur in many aged eukaryotic species and senescent cell types, a gain of heterochromatin is found in oncogene-induced senescent (OIS) cells that form senescence-associated heterochromatin foci (SAHF) [6].

Higher-order chromatin organization has attracted great attention in recent years. With the advent of chromosome conformation capture (3C) methods and high-resolution nuclear microscopy, tremendous progress in our understanding of chromatin organization has been made. At the higher end of chromatin organization, interphase chromosomes form distinct territories called chromosome territories (CTs); small and active chromosomes tend to locate at the center of the nucleus, whereas inactive and heterochromatic regions are located in the nuclear periphery [7,8,9]. Using Hi-C technique, Lieberman-Aiden et al. demonstrated that, in humans, chromatin is spatially segregated into two compartments [10]. Follow-up studies with higher data resolution revealed that individual chromosomes are partitioned into topologically associating domains (TADs), which are highly conserved across cell types and species. At the lower level of chromatin organization, long-range chromatin loops were identified within TADs from super-resolution 3C-based sequencing data [11,12]. The development of 3C-based methods has also shed new light on the chromatin organization changes during aging and cellular senescence. In this review, we discuss recent findings regarding chromatin organization, the factors that regulate it, and how they change during physiological aging, premature aging syndromes, and cellular senescence.

## 2. General Chromatin Architecture

### 2.1. Nucleosome

From linear DNA to the 3D nucleus, chromatin organization is well characterized on both the small scale and the very large scale; however, our understanding of the intermediate levels of chromatin organization remains limited. At the finest scale, DNA is first packaged into nucleosomes, the basic subunit of chromatin. Each nucleosome includes a nucleosome core, a linker DNA, and a H1 linker histone. The structure of the eukaryotic nucleosome is generally conserved: it is composed of 147 bp of DNA coiled around a histone octamer that contains two copies of four core histone proteins, H2A, H2B, H3, and H4 [13,14]. This effectively protects DNA from nucleases and occludes the binding of large protein complexes. It also provides a basic framework for histone modifications, allowing the epigenetic regulation of most biological processes including DNA repair, transcription, and chromatin remodeling [15]. Nucleosome structure is dynamic and functions as a soft thermodynamic barrier to *trans* regulatory elements, rather than as an absolute block. Nucleosomal DNA rapidly unwraps from and rewraps on the surface of the histone octamer, allowing the invasion of DNA binding proteins [16]. Histone protein conformation can also be changed by H2A/H2B dimer splitting [17], H3/H4 tetramer flipping [18], and nucleosome gapping [19]. The chain of nucleosomes linked by linker DNA and linker histone forms the 11 nm chromatin fiber, which has the appearance of “beads on a string” when viewed by electron microscopy [20].

### 2.2. Chromosome and Chromosome Territories

At the highest scale, metaphase eukaryotic chromosomes are condensed and can be visualized under the light microscope. The morphology of metaphase chromosomes has been characterized with well-defined shapes and sizes. In contrast, interphase chromosomes are less condensed and difficult to distinguish by imaging and segmenting. Interphase chromosomes are not randomly positioned in the nuclear space but are generally segregated, with each chromosome forming distinct CTs [21,22]. Interactions between different CTs are limited and restricted to the borders of CTs [23]. The existence of CTs is also supported by Hi-C experiments, where dramatically higher contact frequencies are observed within chromosome than between chromosomes [10].

The human nucleus is functionally compartmentalized [7]. Small chromosomes and those enriched for open and gene-rich regions tend to locate at the center of the nucleus, whereas large chromosomes and those with more heterochromatin are found near the nuclear margins [8]. The genomic regions that are in contact with the nuclear lamina are called lamina-associated domains (LADs). LADs have several typical features of heterochromatin, including low gene density, repressed gene expression, and enrichment for repressed histone marks [24]. LADs help anchor heterochromatic regions to the nuclear lamina, which is essential for the origination of chromosomes inside the nucleus. Recent studies confirmed the peripheral position of heterochromatic CTs by simulation models using chromosome–chromosome interaction data from human lung fibroblasts (IMR90) and embryonic stem cells (ESCs) [25]. Chromosome territories are in regular ellipsoid-like shapes [26], and the ellipsoid-like shape of CT is negatively correlated with gene density in humans, such that gene-poor chromosomes, like chromosome Y, tend to look more like ellipsoid, whereas gene-rich chromosomes, such as chromosome 19, look more irregular [27]. Orsztynowicz et al. studied chromatin in bovine cells and found that irregularly shaped CTs tend to have increased transcription activity [28]. Another example of the correlation between the shape of CT and transcription activity is X chromosome inactivation. Comparisons between active and inactive X chromosomes revealed that the inactive copy exists in a smooth and round shape, whereas the active copy has a more irregular surface [29].

### 2.3. Chromosome Compartmentalization

Multiple lines of evidence suggest that chromosomes are partitioned into different compartments or domains. Lieberman-Aiden et al. defined two compartments in the human genome, compartment A and compartment B, at the megabase scale using Hi-C, with the idea that domains belonging to the same compartment should have more frequent interactions [10]. Chromatin compartments are mathematically defined by the first component of principal components analysis [10]. When linking to the features of chromatin, compartment A represents active, gene-rich regions and is enriched for DNaseI hypersensitive sites and the H3K36me3 mark. In contrast, compartment B mostly represents the heterochromatic domains and overlaps with the lamina-associated domains (LADs) [30]. As confirmed by fluorescent in situ hybridization (FISH), compartment B is more condensed than compartment A, indicated by a closer spatial localization within the nuclear of equidistant probes in compartment B than in compartment A. Because of the specific epigenetic states of the compartments, accurate predictions of chromatin compartmentalization can be made using epigenetic datasets such as DNase hypersensitivity sequencing and DNA methylation microarrays [31].

The existence of compartment A and B have been confirmed at the single-cell level. By multiplexed FISH, these compartments are found to be spatially segregated [32]. Similar spatial segregation was also found in the *Drosophila* genome at the sub-megabase scale. Boettiger et al. (2016) partitioned the *Drosophila* chromosomes into three distinct domain types, i.e., active, inactive, and Polycomb-repressive, according to their epigenetic states. Using super-resolution imaging, they showed that each domain type has a distinct packing density and that Polycomb-repressed domains are spatially excluded from neighboring active domains [33].

More detailed genome contact maps generated by Hi-C revealed that compartments A and B can be broken down into smaller sub-compartments (A1–A2 and B1–B3, respectively) with a median size of 185 kb [11]. Each of the sub-compartments is associated with a specific chromatin state. Both A1 and A2 are gene-rich regions and enriched for H3K36me3, H3K27ac, H3K4me1, and H3K79me3; however, A2 is more enriched for H3K9me3 than A1. Sub-compartment B1 is enriched for H3K27me3 and depleted of H3K36me3. While neither B2 nor B3 is enriched for common histone modifications, B2 and B3 are both enriched in the nuclear lamina, but only B2 is enriched in nucleolus-associated domains, whereas B3 is depleted from the domains.

The partitioning of the genome into compartments A and B is cell type-specific. For instance, during human embryonic stem cell (ESC) differentiation, as much as 36% of the human genome undergoes compartment switching, i.e., transitions from compartment A to B, or vice versa. However, the fraction of the genome that localizes to different compartments is considerably smaller when comparing two differentiated cell types [34]. By examining genome compartmentalization in 21 primary human tissues and cell types, Schmitt et al. demonstrated that the same genomic region is partitioned into a different compartment depending on the tissue examined [35].

### 2.4. Topologically Associating Domains

Chromosome compartments can be further partitioned into smaller domains, called TADs. TADs are the basic unit of chromatin 3D organization. They were first identified from mouse ES cell Hi-C data, and can be visualized in contact maps as highly self-interacting regions [36]. Follow-up studies on chromatin 3D structures revealed several important features of TADs. First, TADs are closed and self-interacting structures, which are insulated from their neighbors by their boundaries. This is reflected in the interaction frequencies, which are much higher within a TAD than they are between TADs. Second, TADs are highly conserved among different cell types and even across species. TADs remain largely unchanged during many biological processes and genetic manipulations including cell differentiation, senescence, and linker histone H1 depletion [34,37,38]. A comparative Hi-C analysis across four mammalian species revealed that TADs are conserved in syntenic sequences [39]. Third, TADs are hierarchical structures. TAD-like structures, called sub-TADs, can be identified within TADs, using high-resolution Hi-C data [11,40]. Fourth, TADs are important functional domains. They provide the environment for gene regulation through interactions between enhancers and promoters. Recent evidence shows that enhancer–promoter interactions within the same TADs tend to have coordinated activities, resulting in coordinated gene expression in the same TADs [34,41,42].

### 2.5. Chromatin Looping

With the development of super-resolution Hi-C and other 3C-based methods such as ChIA-PET, Capture Hi-C, HiChIP, and PLAC-seq, specific long-range interactions between functional loci, or chromatin looping, can be detected [11,43,44,45,46]. Chromatin looping interactions are insulated by TAD boundaries; hence, most of these long-range interactions occur within TADs. Chromatin loops are often found between active promoters, enhancers, and CTCF binding sites [47]. Within the same TAD, super long-range interactions are very rare, except for the *Hox* genes, suggesting that specific 3D interactions are limited by linear distance [48]. Many constitutive enhancer–promoter interactions are pre-established before gene activation. These chromatin loops are mainly mediated by CTCF and conserved across cell types [11,49,50]. In addition to constitutive enhancer–promoter interactions, cell type- or tissue-specific chromatin loops are also found. For example, strong interactions between α-globin promoters and their enhancers are only detected during erythropoiesis, coinciding with the expression of the α-globin genes [51].

## 3. Factors Regulating Chromatin Architecture and Their Roles in Aging and Senescence

### 3.1. CTCF

CTCF binding sites are abundant and widely distributed in bilaterian genomes, but absent in other eukaryotes [52]. Traditionally, CTCF has been considered an insulator, forming a barrier to inhibit heterochromatin spreading [53,54]. Recent 3D genome studies revealed that CTCF actually functions as an architectural protein and plays an important role in the formation of chromatin loops and the regulation of 3D DNA topology [54]. Most CTCF binding sites found at chromatin loop anchors are oriented in a convergent direction, which is thought to be critical for generating chromatin loops [12]. Indeed, several loop extrusion models have been developed on the basis of the convergent orientation of CTCF sites, which can effectively simulate the formation of TADs [55,56,57]. However, a significant number of loop anchors and domain boundaries do not contain CTCF binding sites, indicating that other mechanisms, possibly sequence homology or DNA supercoiling, are also involved in the formation of chromatin loops [49,58]. Indeed, genome-wide depletion of CTCF by small hairpin RNA (shRNA)-mediated knockdown only results in a slight reduction of intra-TAD interactions and has no significant influence on TAD boundaries [59]. Consistent with this, recent studies show that acute CTCF depletion leads to loss of local insulation in the majority of TADs, whereas 20% of TADs and all chromosome compartments are unaffected [60].

CTCF, which has been shown to be reduced during aging or senescence in various models, plays important roles in these processes. The senescence effector p16^INK4a^, which drives the irreversible cell cycle arrest characteristic of senescence, is encoded by the *INK4/ARF* locus, which contains at least three CTCF binding sites [61]. *p16^INK4a^* expression is negatively regulated by CTCF, which drives the formation of compact chromatin loops at its promoter [61]. The high levels of CTCF in proliferating fibroblasts promote *p16^INK4a^* silencing; however, under oncogene-induced senescence, during which CTCF is down-regulated, these loops are disrupted, resulting in high expression of *p16^INK4a^* [61]. CTCF also regulates the relaxation of the imprinted locus (*IGF2*) in aged and senescent cells. In both prostate cells that have undergone replicative senescence and those isolated from aged mice, decreased CTCF levels result in less binding of this protein to the *IGF2* imprinted region [62,63]. Reduced CTCF binding leads to the loss of imprinting and upregulation of *IGF2*, and the effects are more extensive in aged individuals with prostate cancer. This suggests that CTCF-mediated imprinting pattern changes may contribute to aging-related gene expression and cancer progression [62,63]. CTCF is also involved in Cockayne syndrome, a premature aging disease that is caused by mutations in a chromatin remodeling protein called the Cockayne syndrome group B protein (CSB). CSB is an ATP-dependent chromatin remodeler that belongs to SWI2/SNF2 families, with the ability to randomize regularly spaced nucleosomes on chromatin [64]. Cells derived from Cockayne syndrome shows elevated levels of reactive oxygen species (ROS). Under oxidative stress, CTCF and CSB reciprocally regulate each other, with CTCF increasing CSB genomic occupancy at promoter regions and CSB promoting CTCF–DNA interactions [65].

### 3.2. Cohesin

Cohesin is a ring-shaped complex that contains four core subunits, SMC1, SMC3, RAD21, and STAG1, and several cohesin-associated proteins [66]. It regulates many chromatin-associated processes, such as DNA double-strand-break repair, transcriptional regulation through *cis* regulatory elements, and sister chromatid segregation during mitosis [67]. Murayama et al. purified the cohesin complex from fission yeast and showed that it can mediate DNA–DNA tethering by efficiently embracing a second DNA strand after initial DNA loading [68]. Cohesin and CTCF are both important architecture proteins and often co-localize at the CTCF binding sites at TAD boundaries, but their functions in chromatin organization are different [59]. Cohesin, together with its associated protein PDS5, plays a vital role in TAD formation and long-range interactions [69], while CTCF is largely dispensable for these processes, as discussed above. Cohesin can also be recruited to the enhancer and active promoters by Mediator and promote the formation of cell-type specific loops independently of CTCF [70]. Depletion of the cohesin complex leads to the loss of DNA interaction domains, and some of the interactions can be rapidly reconstructed after the restoration of RAD21 [71]. A recent study of long-range chromatin loops revealed that H3K4me1, which is deposited on enhancers by the histone methyltransferases MLL3 and MLL4, helps drive cohesion-mediated enhancer–promoter interactions [72].

Because of the function of the cohesin complex, it is particularly essential to prevent genome instability, which is one of the hallmarks of aging [1]. As with CTCF, cohesin levels decrease with age in various models. In budding yeast, genomic instability at ribosomal DNA regions increases during replicative aging; this is accompanied by the loss of cohesin in this and other genomic regions, including centromeres [73]. Cohesin is also implicated in reproductive aging in *Drosophila* and mammals, where the fidelity of meiotic chromosome segregation significantly declines with age. In *Drosophila*, a significant increase of meiotic segregation errors was observed in aged *Drosophila* oocytes when SMC1 is reduced [74]. In mammalian species, it has been reported that cohesin is a key molecular link between the decline of meiotic segregation and female aging [75,76].

### 3.3. Mediator and Other Factors

Mediator is a large, multi-subunit complex essential for regulating transcription initiation and elongation [77]. It is also important for maintaining chromatin architecture and long-range enhancer–promoter chromatin looping. Chromatin immunoprecipitation (ChIP)-seq data showed that MED2, a subunit of Mediator, localizes to the genome either alone or together with cohesin, but rarely overlaps with CTCF in the absence of cohesin [70,78]. Mediator and cohesin often anchor cell type-specific looping interactions, whereas CTCF and cohesin are responsible for maintaining constitutive long-range chromatin loops, as reflected by their genome-wide localizations [78]. Knockdown of Mediator causes chromatin looping disruption and aberrant gene expression, but no effects on TAD boundaries, indicating that it functions in local chromatin looping but does not contribute to higher-order chromatin structure [40,79,80]. At the nucleosome level, Mediator maintains nucleosome-free regions at gene promoters, which is required for the assembly of the pre-initiation complex [81], and interacts with chromatin remodeling complexes such as SWI/SNF and CHD1 to facilitate nucleosome redistribution during transcription activation [82,83].

Polycomb proteins and non-coding RNAs are also implicated in 3D chromatin organization. In the mammalian genome, depletion of Polycomb subunits results in the disruption of chromatin loops around important developmental genes, such as *Hox* gene cluster [48,84]. Polycomb proteins are also involved in the repression of *p16^INK4a^* in young individuals [85], and the high expression of *p16^INK4a^* in old tissues is partially derived from the decline of Polycomb protein BMI1, which is a potent repressor of *p16^INK4a^* [86]. In addition, it has been reported that Polycomb proteins, such as BMI1, EZH2, and SUZ12, were downregulated in senescent human multipotent stem cells [87]. Non-coding RNAs directly bind to CTCF and Mediator to regulate chromatin looping. For example, depletion of enhancer RNAs (eRNAs), which interact with the Mediator subunit MED12, abolishes chromatin looping between the enhancer and the promoter. The recently identified *ThymoD* non-coding RNA promotes DNA demethylation at CTCF binding sites and facilitates the formation of cohesion-mediated chromatin loops between the promoter of *Bcl11b* and its enhancer [88].

Numerous studies reported that noncoding RNAs are associated with lifespan. They can either be products or regulators of aging and cellular senescence. In yeast, the expression of ncRNAs from the rDNA locus, which is repressed by Sir2 under normal conditions, is significant upregulated in old individuals [73]. In addition, mutations that silence the expression of the rDNAs result in lifespan extension and make SIR2 dispensable for lifespan extension [89]. In *Caenorhabditis elegans*, multiple miRNAs, including *lin-4*, *mir-71*, and *mir-245*, promote lifespan [90]. In mammals, multiple miRNAs are downregulated in fat tissue during aging, which can largely be prevented by caloric restriction [91].

## 4. Chromatin Architecture in Senescence and Aging

**Figure 1:**
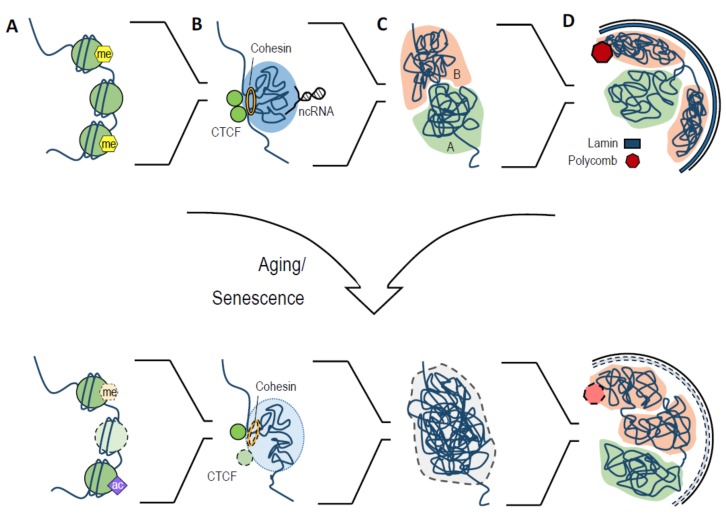
An overview of chromatin structural changes during aging and senescence. (**A**) Chromatin structural changes at the nucleosome level, including global loss of core histones, epigenetic changes, and nucleosome repositioning. (**B**) Topologically associating domains (TAD) structure and anchor proteins changes. The levels of TAD boundary anchor proteins such as CTCF and cohesin are reduced during aging, with altered local chromatin interaction changes. (**C**) Chromosome compartment changes. Global loss of chromosome compartment occurs at late passages of Hutchinson–Gilford progeria syndrome (HGPS) fibroblasts. A small subset of chromosome compartments go through compartment switching in premature aging disorders and senescence. (**D**) Large-scale chromatin rearrangements. The reduction of lamin proteins during aging and senescence leads to the detachment of lamina-associated domains (LADs) from the nuclear lamina. Depletion of Polycomb proteins, which are also involved in the genome 3D organization, is observed in senescent cells. Me: methylated histone; ac: acetylated histone; ncRNA: non-coding RNA.

Cellular senescence and aging are related but distinct biological process that occur on different scales. Growing evidence shows that cellular senescence has a role in aging and is one of the major causes of age-related diseases [92,93,94,95]. Senescent cells secrete a host of mostly pro-inflammatory cytokines (called the senescence-associated secretory phenotype, or SASP), which causes tissue dysfunction and promotes oncogenesis due to chronic inflammation during aging [96]. Consistent with a role for senescent cells in organismal aging, elimination of such cells extends mouse healthspan, reduces several age-associated conditions, and improves resistance to age-related diseases [97,98]. During aging and senescence, there are dramatic changes in the chromatin landscape at different levels of organization, from those in linear structures, such as histone and nucleosome, to those in spatial structures, such as chromatin compartments and TADs (Figure 1).

### 4.1. Global Histone Changes during Aging

Global loss of core histone proteins, which partially contributes to the increase of genome instability, occurs during aging and senescence [99] (Figure 1A). Compelling evidence shows that histone protein loss is associated with aging in many species, including yeast, worms, and mammals [99,100,101,102]. In budding yeast, at least, the loss of histones is associated with global transcriptional upregulation, which may explain the observed increase in histone transcripts, despite the decline in their protein levels [99,100]. Overexpression of histones or deletion of the Hir complex, which results in elevated histone proteins, extends yeast lifespan [100]. In worms, deletion of *set-26* extends lifespan and moderately attenuates the decrease in histone H3 protein levels [101]. SET-26 is a large protein with a SET domain and a PHD-zinc finger, but little is known about the function of SET-26 and its exact mechanisms behind longevity promotion. In mammals, a reduction in histone protein levels has been reported in senescent cells, and several molecular mechanisms have been proposed to explain how this occurs. O’Sullivan et al. reported that in old IMR90 fibroblasts, chronic damage stress from shortened telomeres causes reduced histone H3 and H4 biogenesis, resulting in decreased protein levels [102]. In addition to reduced histone synthesis, the decrease in global histone levels could also be partly caused by cytoplasmic chromatin processing. During replicative and oncogene-induced senescence, chromatin fragments are extruded from the nucleus, where they are targeted and processed by the autophagy/lysosomal pathway. This leads to the depletion of histone proteins and may contribute to the stability of senescence [103].

### 4.2. Histone Modification Changes with Aging

In addition to core histone protein levels, histone modifications also play important roles in regulating aging and chromatin structures (Figure 1A). The changes of histone modifications during physiological aging and premature aging syndromes are usually accompanied by the loss or redistribution of heterochromatin, which is associated with the alternation of global gene expression and increased genome instability [2,4]. Zhang et al. reported a global loss of H3K9me3 and large-scale heterochromatin disorganization in mesenchymal stem cells (MSCs) isolated from patients with Werner syndrome, a premature aging disorder caused by WRN deficiency [104]. In line with this, co-immunoprecipitation (Co-IP) experiments revealed that WRN interacts with H3K9me3 methyltransferase SUV39H1, heterochromatin protein HP1α, and lamina–heterochromatin anchoring protein LAP2β [104]. Additionally, a comparison between MSCs derived from young and old human individuals showed a similar reduction in H3K9me3 and heterochromatin disorganization. Significantly, the level of WNR protein also decreases with age, accompanied by downregulation of H3K9me3, SUV39H1, HP1α, and LAP2β [104]. As in Werner syndrome, heterochromatin loss is also observed in fibroblasts derived from Hutchinson–Gilford progeria syndrome (HGPS) patients, as shown by the decreased levels of H3K9me3, HP1α, H3K27me3, and H3K27me3 methyltransferase EZH2 [105,106,107]. Interestingly, Liu et al. reported an increased level of H3K9me3 and its methyltransferase *Suv39h1* in progeroid cells, which was associated with a DNA repair deficiency in heterochromatin regions. They also reported that depletion of *Suv39h1* in progeroid mice prolongs their lifespan and suggested that increased H3K9me3 in these mice may promote heterochromatin compaction and obstruct the access of DNA repair factors [108]. Together, these studies suggest that, in mammals, alterations in the level of H3K9me3 occur during aging and premature aging disorders, which leads to the redistribution of heterochromatin, decreased DNA repair ability, and increased genome instability.

Alterations in histone modifications are also implicated in lifespan regulation in other organisms. In budding yeast, the levels of the histone deacetylase Sir2 decrease during replicative aging, which causes an increase in H4K16 acetylation [109]. Overexpression of Sir2 or deletion of the histone acetyltransferase Sas2 extend lifespan, suggesting that H4K16ac has a causative effect on budding yeast aging [109]. Sir2 also extends yeast lifespan by repressing the formation of extrachromosomal rDNA circles (ERC), and the accumulation of ERC causes aging [110]. Deletion of Sir2 greatly shortens yeast lifespan, with increased rDNA recombination and ERC formation [111]. A recent study revealed that H3K36me3 plays important roles in transcription fidelity and yeast replicative lifespan [112]. Depletion of H3K36me3 during yeast aging results in increased cryptic transcription and shortened lifespan, whereas deletion of the H3K36 demethylase *Rph1* reduces the level of cryptic transcription and extends lifespan [112]. In *C. elegans*, lifespan can be extended by reducing H3K4me3 and increasing H3K27me3 [113,114,115]. The level of H3K27me3 significantly decreases in somatic cells during aging [114]. Depletion of UTX-1, a histone demethylase for H3K27me3, increases the global levels of H3K27me3 and extends worm lifespan [114]. Similarly, knockdown of H4K4me3 methyltransferase subunits decreases the global level of H3K4me3 and promotes longevity. Conversely, knockdown of a H3K4me3 demethylase increases the global level of H4K4me3 and shortens lifespan [115]. In *Drosophila*, aging is associated with a loss of H3K9me3 in heterochromatin and a gain of H3K4me3 and H3K36me3 [116].

### 4.3. Nucleosome Remodeling during Aging

Nucleosome structure is relatively stable, whereas the distribution of nucleosomes along chromatin is variable and can be regulated by ATP-dependent nucleosome remodeling complexes, leading to either more compact or more accessible chromatin. Deletion or inactivation of the chromatin remodeler ISW2 promotes longevity in budding yeast, in a TOR signaling-independent manner [117]. Deletion of ISW2 derepresses genes involved in stress response pathways and activates the homologous recombination DNA damage repair pathway. In line with this, the transcriptome and nucleosome positioning profiles of ISW2 deletion strain are similar to those of cells under caloric restriction, in which the activation of stress response pathways also occurs [117]. RAD51, which is involved in homologous recombination, is significantly upregulated in the *isw2* deletion strain, and overexpression of RAD51 extends yeast lifespan [117], confirming that the nucleosome remodeler ISW2 promotes yeast longevity through a DNA repair pathway. DAF-16/FOXO is a key transcription factor downstream of many important pathways, including insulin/IGF-1 signaling pathway, TOR signaling, and AMPK pathway [118]. The chromatin remodeler SWI/SNF is required for the activation of DAF-16/FOXO target genes during longevity promotion in worms [118]. It interacts with DAF-16/FOXO and binds to the promoters of DAF-16/FOXO target genes in order to activate them by chromatin remodeling. In agreement with this, inactivation of the SWI/SNP core subunits by RNAi fully suppresses the lifespan extension induced by DAF-16/FOXO.

Another ATP-dependent chromatin remodeling complex known to be involved in aging is the nucleosome remodeling and deacetylase (NURD) complex. The protein levels of NURD subunits RBBP4 and RBBP7 are reduced in HGPS skin fibroblasts; this is associated with lower levels of HP1, indicating global chromatin defects [119]. Primary cells derived from older patients show a similar reduction of RBBP4 and RBBP7, suggesting that the loss of NURD is associated with both premature aging and physiological aging. Conversely, depletion of the NURD subunit LET-418/Mi2 promotes lifespan and increases stress resistance in both *Drosophila* and *Arabidopsis* [120]. However, depletion of other NURD subunits does not result in longevity promotion, suggesting that LET-418 may regulate longevity in a NURD-independent manner [120]. In summary, these studies reveal the involvement of NURD in aging regulation, although the exact molecular mechanism by which it contributes to this process is still unclear.

### 4.4. Chromatin Conformation Changes during Aging and Senescence

As mentioned above, SAHFs are a special senescence-associated structure that only form in specific types of senescent cells [121]. They are prominent in acute senescence models, such as OIS, whereas in senescent cells induced by chronic stress, such as replicative stress or progeroid syndromes, SAHFs are infrequent, and cells usually display a significant loss of heterochromatin. [6,105,122]. The formation of SAHFs has been reviewed in detail [4]. Briefly, chromatin is initially condensed by the HUCA histone chaperone complex, then proteins such as HP1, macro H2A, and HMGA, are recruited to the condensed region. The eventual formation may be triggered by the loss of nuclear lamina, which breakdowns the heterochromatin organization. Consistently, Chandra et al. revealed that SAHFs are multilayer structures with constitutive heterochromatin at the center of chromosomal territories [123]. The formation of SAHFs is accompanied by spatial repositioning of pre-existing histone marks such as H3K9me3 and H3K27me3 [123]. However, depletion of the SAHF architectural protein HMGA1 does not alter the chromatin landscapes of repressive histone marks. Also, OIS cells with depleted H3K9me3 and H3K27me3 still form SAHFs. Altogether, SAHFs are thought to be a protective structure that will be formed under senescent-related chromatin defects including normal heterochromatins disruption, histone loss, and increased transcriptional noise.

The formation of SAHFs is one example of the disassociation between the nuclear lamina and chromatin during aging and senescence. The nuclear lamina consists two types of lamins (A and B) and associated proteins. Mutations or depletion of lamin proteins leads to severe changes, including abnormal nuclear morphology, DNA damage, and chromosomal aberrations [3]. For example, HGPS is caused by a mutation that activates a cryptic splice site in lamin A, resulting in the accumulation of mutated lamin A. The mechanisms behind HGPS also apply to normal physiological aging [105]. The same lamin A product produced by HGPS-inducing mutations is found in normal old individuals, who have similar nuclear defects as those in HGPS patients. Inhibition of the cryptic splice site reverses age-related nuclear defects, confirming the causative effect of the lamin A mutation in HGPS [105]. Lamina-associated domains (LADs) contain gene-poor and heterochromatic regions that interact with the nuclear lamina. The loss of nuclear lamina components causes the detachment of LADs from the nuclear envelope, leading to the redistribution of heterochromatin from the nuclear periphery to the interior [124]. The expression of Lamin B1 is reduced in HGPS cells, replicative senescent fibroblasts, OIS cells, and cells with high levels of chronic DNA damage [125,126,127]. Furthermore, Shimi et al. reported that, in WI-38 fibroblasts, knockdown of lamin B1 slows cell growth and triggers premature senescence, while overexpression of lamin B1 promotes proliferation and delays the onset of senescence [126]. Taken together, these results reveal the essential roles of lamin proteins in chromatin structures, chromosome territories, and longevity.

Although the development of the Hi-C method greatly improved our understanding of normal genome architecture, studies that focus on chromatin higher-order structure changes during aging are limited. Overall, changes in 3D chromatin structure, such as local interaction frequencies (Figure 1B) and chromosome compartmentalization (Figure 1C), are found in the late passages of HGPS cells and different types of senescent cells [128,129,130]. In the aging field, the first Hi-C analysis was conducted in HGPS fibroblasts [128]. Late-passage HGPS fibroblasts have a catastrophic global loss of chromosome compartments, reduced H3K27me3 and EZH2, and disassociation of heterochromatin from the nuclear lamina [128] (Figure 1D). The dramatic loss of chromosome compartments was also confirmed by high-resolution microscopy imaging. In the regions that could still be assigned to compartments, a large number underwent compartment switching with cell passaging [128]. Compartments switching has been linked to gene expression alternation during ESC differentiation [34], so it would be particularly interesting to see the effect that the impressive chromosome compartmentalization changes that occur in late-passage HGPS fibroblasts have on gene expression.

A Hi-C experiment has also been conducted on Ras-induced senescent cells (a type of OIS), most of which contain SAHFs. No global chromosomal interaction pattern changes were observed when comparing proliferating and senescent cells. However, local interactions were reduced in constitutive heterochromatin, accompanied by the formation of specific long-range interactions with other repressed regions [37]. The authors linked the interaction changes to the formation of SAHFs, as they form through the redistribution of constitutive heterochromatin [123]. A comparison between ESCs, somatic cells, and OIS cells revealed that local interactions are progressively lost with declining differentiation ability, suggesting that senescence may be an endpoint of chromosome remodeling during differentiation [37].

Similar to OIS cells, the overall genomic conformation is largely unchanged in cells that have undergone replicative senescence (RS cells); chromatin compartmentalization and TAD boundaries are generally conserved in proliferating, quiescent, and RS cells. However, a global gain of short-range interactions and a loss of long-range interactions were found in RS cells [130]. By measuring genetic distances, chromosome volumes, and chromatin accessibility, the authors concluded that the global interaction changes were caused by the compaction of chromosome arms. Although chromatin compartments are largely conserved, compartment switching occurred in a small subset of the genome during replicative senescence [130]. As expected, the expression of the genes located within these regions generally follows the direction of the compartment switch, confirming the previous finding that chromosome architecture regulates gene expression.

## 5. Concluding Remarks

The development of Hi-C methodologies and high-resolution nuclear imaging techniques are transforming our understanding of chromatin structure and 3D genome organization. How chromatin organization influences aging and is altered by it and the related process of cellular senescence is only beginning to be explored. Decreased histone protein levels and changes in the abundance and distribution of particular histone modifications contribute to the opening of chromatin at the nucleosome level with age and senescence. This, in conjunction with the activity of chromatin remodeling enzymes, contributes to transcriptional dysregulation and, in some instances, genome instability. Consistent with the opening of chromatin, there is a loss of constitutive heterochromatin during aging and in progeroid syndromes. Heterochromatin loss is also associated with the disruption of LAD–nuclear lamina interactions, which potentially have profound effects on nuclear organization and chromatin compartmentalization. However, there have only been a few explicit studies on higher-order chromatin changes during aging and senescence; they suggest that, in most cases, such structures are overall largely preserved. TADs, the basic unit of chromatin organization, are largely intact, though there is a reduction in intra-TAD chromatin interactions. Likewise, the genome is still partitioned into compartments A and B, though compartment switching does occur both in HGPS and during cellular senescence. This compartmentalization is even maintained in cells that have undergone OIS and have formed SAHFs, in which the normally peripheral heterochromatin is localized more centrally, indicating drastic nuclear reorganization.

The picture of age-associated changes in genomic organization is only beginning to emerge, and many critical questions remain unanswered. While key regulators of chromatin higher-order structure, including CTCF and cohesin, are known to decrease with age, how this affects both aging and chromatin structure is largely unknown. Chromatin looping mediated by CTCF, cohesin, and Mediator is essential for proper gene regulation, which is known to be disrupted during aging; however, age-associated changes in sub-TAD-level chromatin looping have barely begun to be explored, probably in part because of the challenges of generating enough sequencing data to obtain the needed resolution. The evidence currently suggests that, in many cases, many of the chromatin organization changes will be found at this level, though we clearly need to examine the 3D genome structure in additional aging models, particularly in primary tissue samples. Another area that deserves more attention is the role of LADs and the nuclear lamina in regulating aging, gene expression, nuclear organization, and the formation of SAHFs. Ultimately, understanding the interplay between different levels of chromatin organization, from how histone modifications contribute to chromatin looping and promote spatial segregation of heterochromatin within the nucleus to how chromatin compaction impacts short-range loop formation and gene expression, will be instrumental in unraveling the mechanisms that drive aging.
